# Supplementary material for: Systematic computer-aided disulfide design as a general strategy to stabilize prefusion class I fusion proteins
Source: Front Immunol. 2024 Jul 24;15:1406929. doi: 10.3389/fimmu.2024.1406929 (PMC11303214; doi:10.3389/fimmu.2024.1406929)
Supplement: Supplementary file 1 [file DataSheet_1.pdf]

Figures

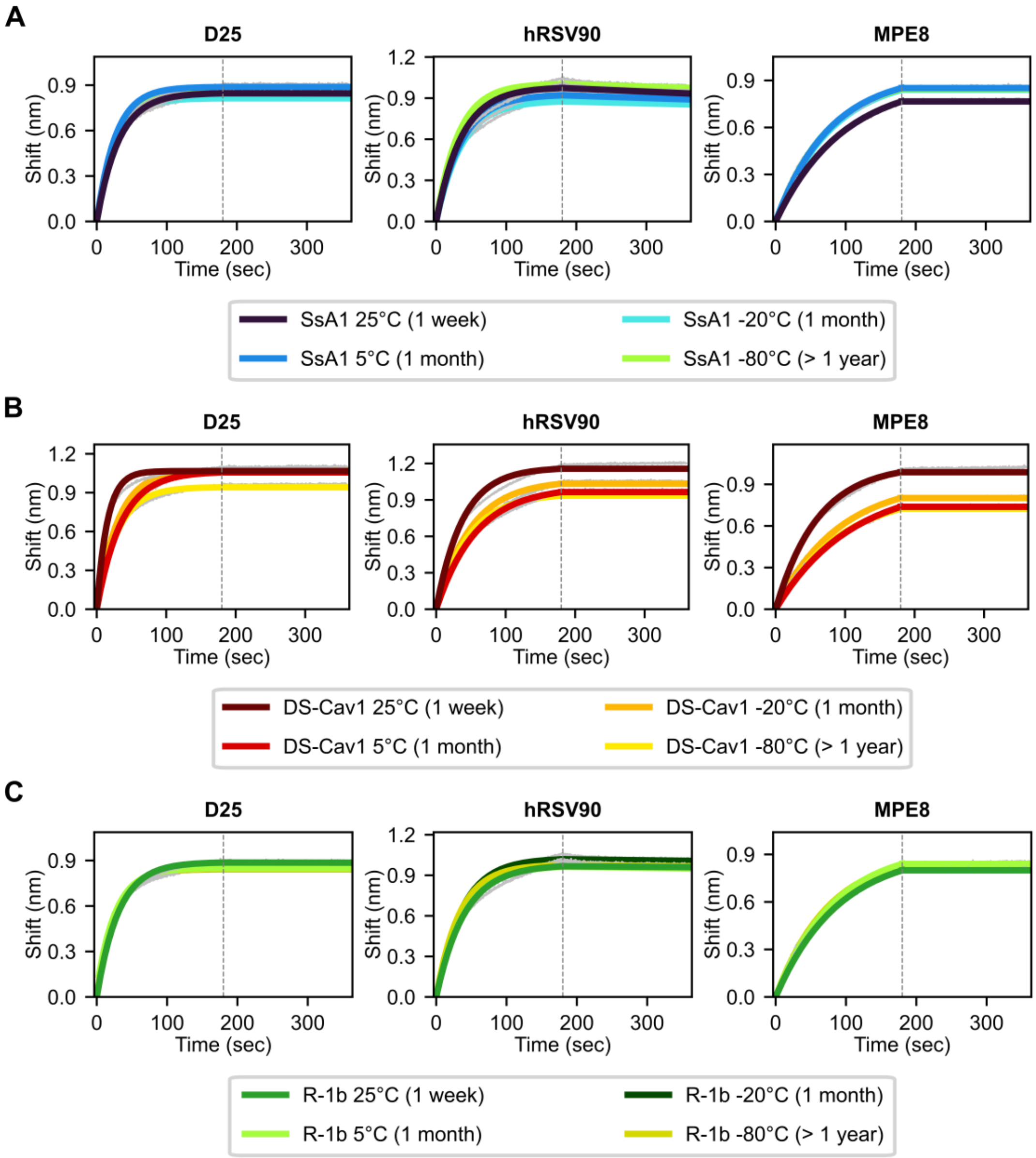

Supplementary Figure 1. Binding of variants **(A)** SsA1, **(B)** DS-Cav1, and **(C)** R-1b to prefusion-specific antibodies after exposure to various storage conditions. Plotted values correspond to binding at the highest protein concentration (200nM). Binding data are shown in grey, while the best fits to a 1:1 binding model are shown in distinct colors. The

end of the association time is delimited with a dotted line. Binding constants are shown in Supplementary Table 2.

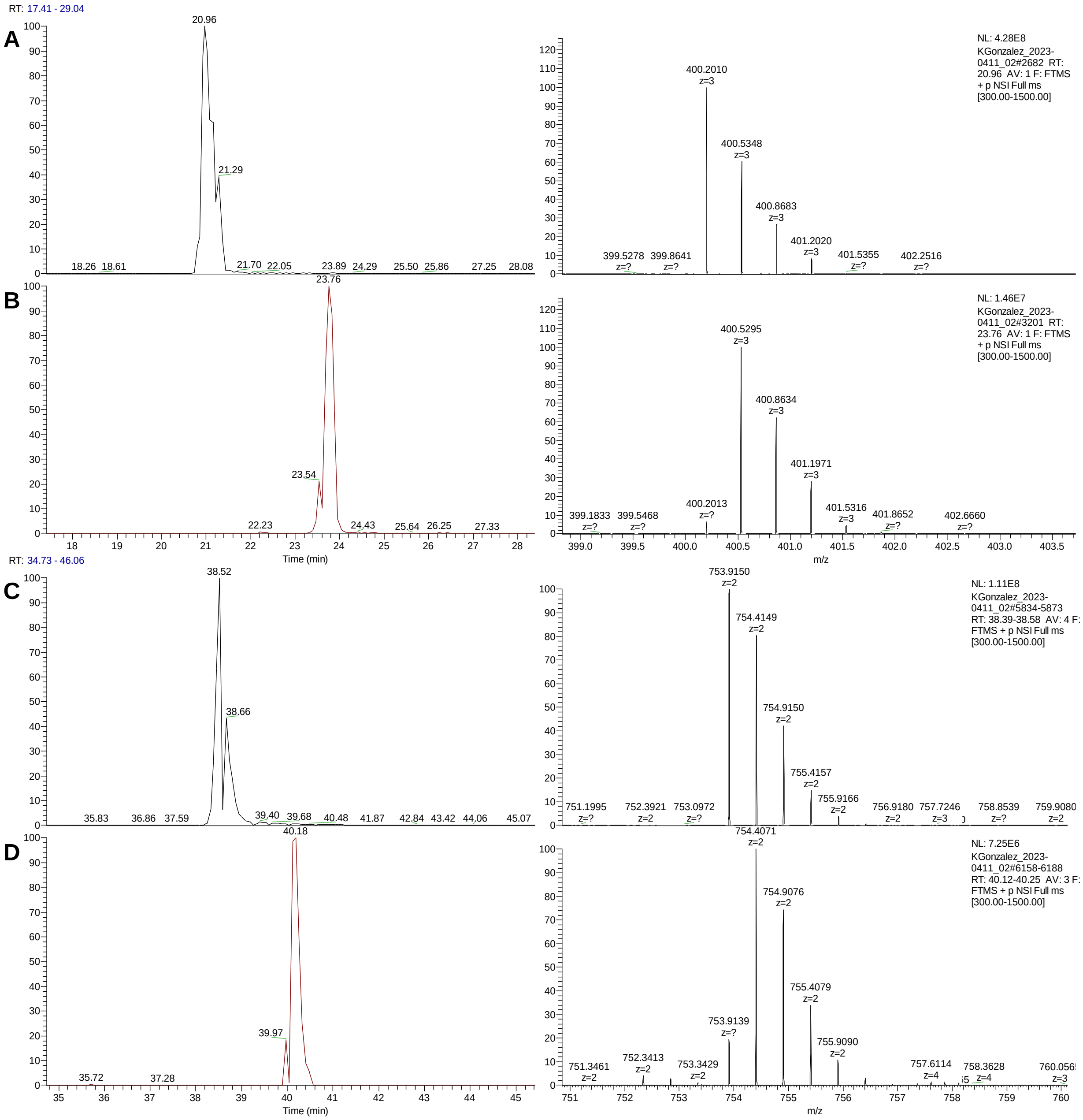

Supplementary Figure 2. Tandem liquid chromatography mass spectrometry (LC-MS/MS) analysis of SsA1 peptides containing cysteine 157 (<u>C</u>LHLEGEVNK) and cysteine 183 (AVVSLS<u>C</u>GVSVLTSK). The SsA1 protein was treated with two alkylation procedures to distinguish free cysteine residues from disulfide-bonding cysteines. Alkylation with iodoacetic acid (IAA) was used to attach carboxymethyl groups to free cysteines while alkylation with iodoacetamide (IAM) was used to label disulfide-bonding cysteines with carbamidomethyl groups, after proper reduction of the disulfide bond. **(A)** IAM-treated (ion m/z 400.2010) and **(B)** IAA-treated (ion m/z 400.5295) spectrums of

peptide containing cysteine 157 (<u>C</u>LHLEGEVNK). **(C)** IAM-treated (ion m/z 753.9150) and **(D)** IAA-treated (ion m/z 754.4071) spectrums of peptide containing cysteine 183 (AVVSLS<u>C</u>GVSVLTSK). Left panels depict ion chromatograms for two types of ions (IAM and IAA treatments) and right panels show the mass spectrum of each sample. Although both peptides were slightly alkylated during the IAA treatment (free-cysteine labeling) as shown in (B) and (D), the base peak intensity of the IAM treatments, as determined by the normalization level (NL) value, evidenced the predominance of disulfide-bonding cysteines. The NL values for IAM and IAA alkylation of peptide <u>C</u>LHLEGEVNK were 4.28E8 and 1.48E7, respectively, while peptide AVVSLS<u>C</u>GVSVLTSK showed 1.11E8 and 7.25E6, respectively.

#1	b^+	b^{2+}	Seq.	y^+	y^{2+}	#2
1	161.03793	81.02260	C-Carbamidomethyl			10
2	274.12199	137.56463	L	1038.55783	519.78256	9
3	411.18090	206.09409	H	925.47377	463.24052	8
4	524.26496	262.63612	L	788.41486	394.71107	7
5	653.30756	327.15742	E	675.33080	338.16904	6
6	710.32902	355.66815	G	546.28820	273.64774	5
7	839.37161	420.18945	E	489.26674	245.13701	4
8	938.44003	469.72365	V	360.22415	180.61571	3
9	1052.48296	526.74512	N	261.15573	131.08150	2
10			K	147.11280	74.06004	1

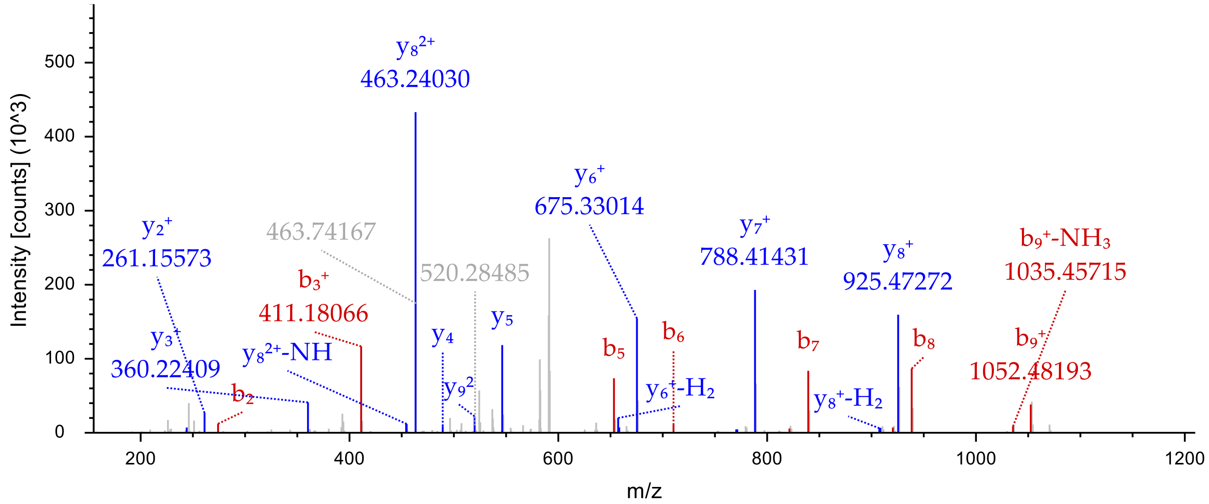

Supplementary Figure 3. Fragmentation spectrum of ion containing SsA1 cysteine 157 (<u>C</u>LHLEGEVNK) labeled with carbamidomethyl (IAM treatment). On display is shown the spectrum of +2 charge state product ion, with monoisotopic m/z: 599.79796 Da (+0.08 mmu/+0.14 ppm), MH+: 1198.58865 Da, and retention time: 21.5271 min. The

peptide was identified with Sequest HT (v1.17); XCorr:3.37, and a fragment match tolerance of 0.02 Da. Fragments used for search were y-H_2O; y-NH_3; b; b-H_2O; b-NH_3; y.

#1	b^+	b^{2+}	Seq.	y^+	y^{2+}	#2
1	72.04439	36.52583	A			15
2	171.11280	86.06004	V	1435.78249	718.39488	14
3	270.18122	135.59425	V	1336.71408	668.86068	13
4	357.21325	179.11026	S	1237.64566	619.32647	12
5	470.29731	235.65229	L	1150.61364	575.81046	11
6	557.32934	279.16831	S	1037.52957	519.26842	10
7	717.35999	359.18363	C-Carbamidomethyl	950.49754	475.75241	9
8	774.38145	387.69436	G	790.46689	395.73709	8
9	873.44986	437.22857	V	733.44543	367.22635	7
10	960.48189	480.74458	S	634.37702	317.69215	6
11	1059.55031	530.27879	V	547.34499	274.17613	5
12	1172.63437	586.82082	L	448.27657	224.64193	4
13	1273.68205	637.34466	T	335.19251	168.09989	3
14	1360.71408	680.86068	S	234.14483	117.57605	2
15			K	147.11280	74.06004	1

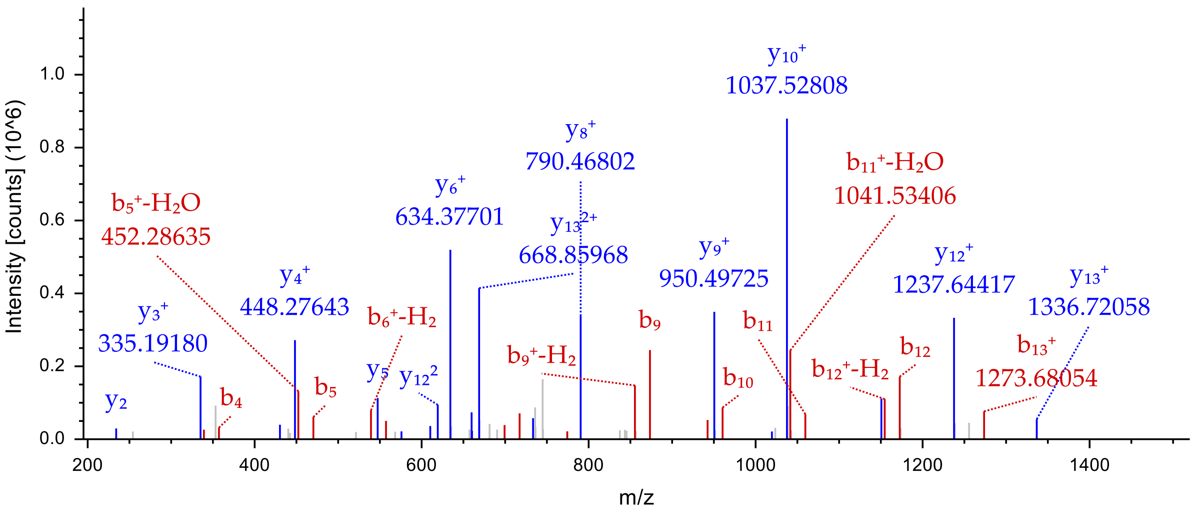

Supplementary Figure 4. Fragmentation spectrum of ion containing SsA1 cysteine 183 (AVVSLSCGVSVLTSK) labeled with carbamidomethyl (IAM treatment). On display is shown the spectrum of +2 charge state product ion, with monoisotopic m/z: 753.91394 Da (+0.5 mmu/+0.66 ppm), MH+: 1506.82061 Da, and retention time: 38.9540

min. The peptide was identified with Sequest HT (v1.17); XCorr:3.69, and a fragment match tolerance of 0.02 Da. Fragments used for search: y-H_2O; y-NH_3; b; b-H_2O; y.

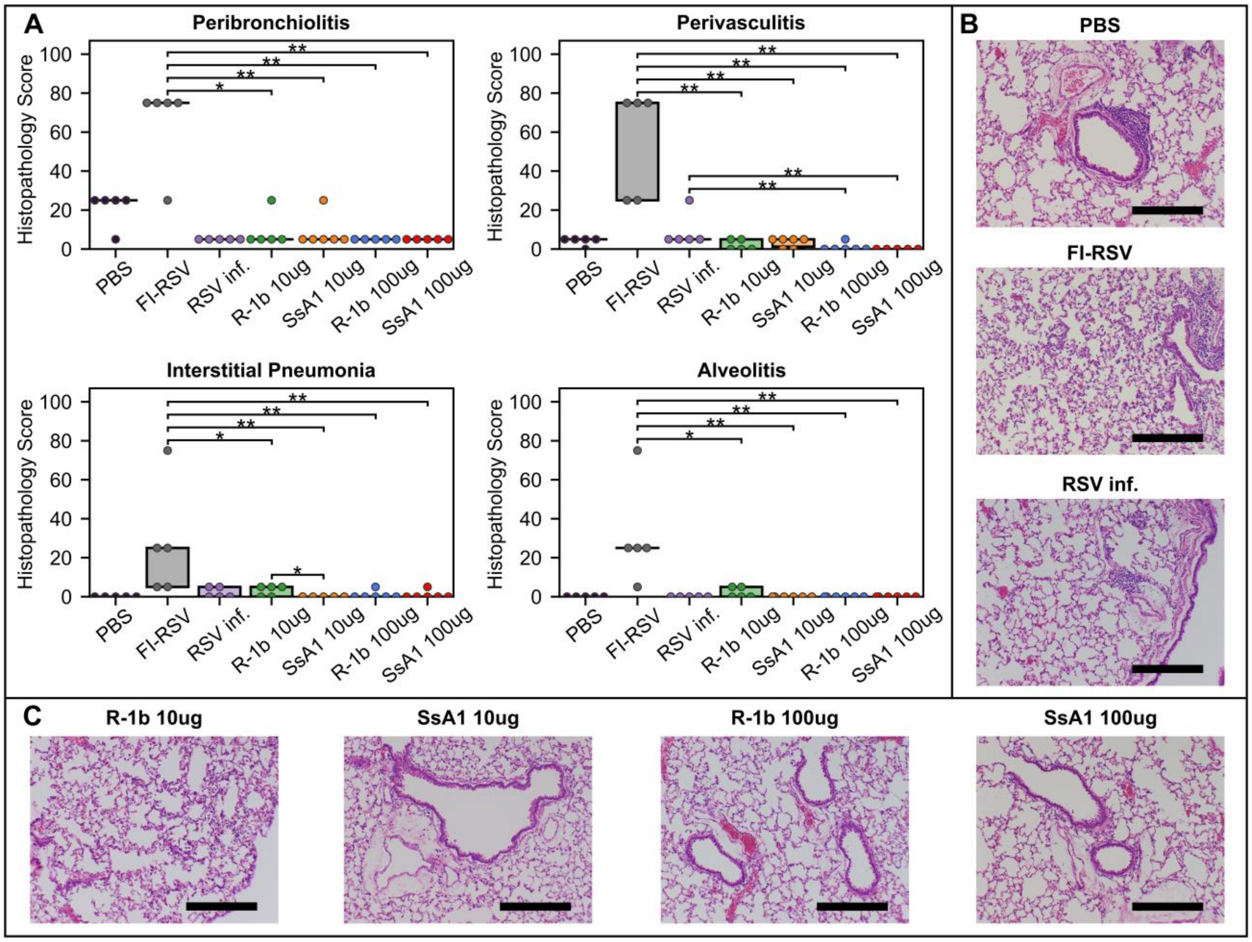

Supplementary Figure 5. Pulmonary histopathology following RSV challenge. (A) Pulmonary inflammatory scores evaluating peribronchiolitis, perivasculitis, interstitial pneumonia, and alveolitis. Control experiments corresponded to a mock vaccination with PBS, vaccination with formalin-inactivated RSV (FI-RSV), and a primary infection with RSV A/A2 on day 0 (RSV inf.). The distribution of the data is shown in boxplots, where inflammatory scores from each animal are represented by circles. All boxplots show the median as a central line, lower and upper quartiles as the box limits, and minimum to maximum values as whiskers. Pairwise statistical analyses were performed with a Mann-Whitney U test. *, $p \leq 0.05$; **, $p \leq 0.01$. **(B)** Representative photomicrographs (100X) of lung sections from control animals, and **(C)** animals vaccinated with R-1b or SsA1. The FI-RSV photomicrograph reveals significant interstitial pneumonia and alveolitis, whereas PBS and RSV-infected examples show mild peribronchiolitis and perivasculitis, respectively. The lung section from the animal vaccinated with 10 µg of R-1b exhibits mild

interstitial pneumonia and alveolitis. The remaining photomicrographs evidence normal histology from the peribronchiolar and perivascular view. Black bars indicate 500 µm.

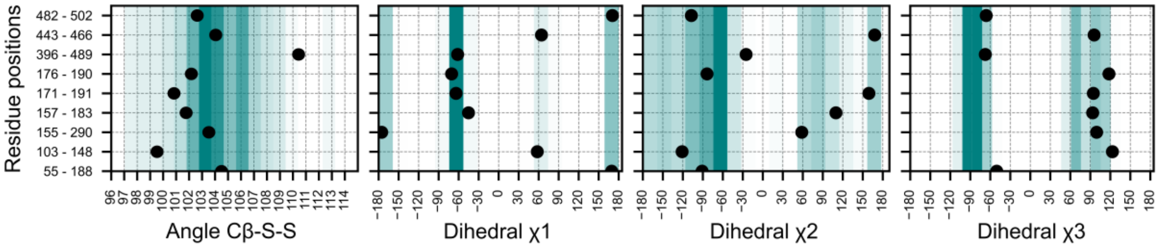

Supplementary Figure 6. Geometry assessment of identified potential disulfides in R-1b including the fusion peptide region. Panels present the bond angles of the newly identified potential disulfides. The background color scales in each plot represent the frequency of each bond angle, according to a set of 300 high-resolution structures (<1.5Å). Darker colors indicate angles that were more frequent in this reference set, while lighter colors represent angles of low frequency.

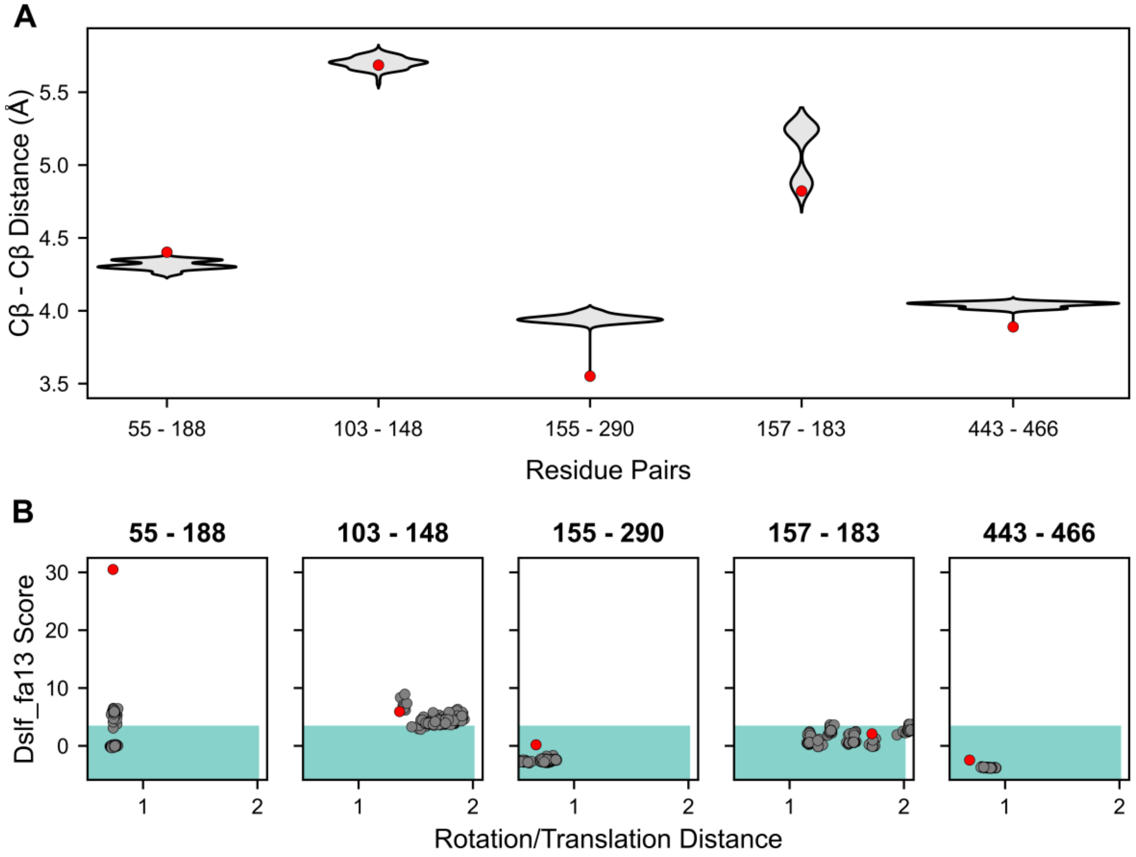

Supplementary Figure 7. Geometric variations across different R-1b conformations. The parameters Cβ-Cβ distance, dslf_fa13 score, and rotation/translation distance were used to determine which pair of residues could form a

disulfide bond. While these parameters were assessed for every pair of residues in the protein, the plots displayed focus on exemplary pairs that have been experimentally proven to stabilize the prefusion state through disulfide introduction. These positions include those identified in this study (55-188 (SsA2), 157-183 (SsA1), 443-466 (SsB1)) as well as others identified by our methodology but experimentally tested elsewhere (155-290 [1] and 103-148 [2]). **(A)** Cβ-Cβ distances distribution for selected pairs of residues across an ensemble of R-1b conformations. Violin plots represent data distribution, with red circles featuring data points from the original crystal structure. **(B)** Variations in dslf_fa13 score and rotation/translation distances for selected pairs of residues following cysteine mutations and backbone relaxation. Gray circles represent individual data points across an ensemble of R-1b conformations, while red circles indicate data points from the original crystal structure. Green-shaded areas highlight the accepted values for potential disulfide bond formation; only disulfides within this range were selected for further analysis. The top of each panel specifies the disulfide pair being analyzed.

Supplementary Table 1. Binding kinetics of RSV F variants after heat treatment, measured by bio-layer interferometry.

Protein Variants	Assay temperature (°C)	Antibodies											
		D25			hRSV90			AM14*			MPE8		
		koff (1/s)	kon (1/Ms)	KD (M)	koff (1/s)	kon (1/Ms)	KD (M)	koff (1/s)	kon (1/Ms)	KD (M)	koff (1/s)	kon (1/Ms)	KD (M)
SsA1	RT	N/A	1.79E+05	N/A	1.06E-04	1.28E+05	8.28E-10	5.41E-02<br>2.16E-03	1.79E+02<br>6.16E+05	3.03E-04<br>3.51E-09	N/A	4.80E+04	N/A
		N/A	1.72E+05	N/A	8.39E-05	1.26E+05	6.65E-10	3.22E-02<br>N/A	3.57E+02<br>8.91E+06	9.01E-05<br>N/A	N/A	5.73E+04	N/A
	65	N/A	1.72E+05	N/A	2.48E-05	1.01E+05	2.46E-10	N/A	N/A	N/A	N/A	2.61E+04	N/A
		N/A	1.75E+05	N/A	1.44E-05	1.02E+05	1.42E-10	N/A	N/A	N/A	N/A	3.08E+04	N/A
	70	N/A	3.59E+04	N/A	N/A	5.15E+04	N/A	N/A	N/A	N/A	N/A	1.98E+02	N/A
		N/A	2.72E+04	N/A	N/A	5.51E+04	N/A	N/A	N/A	N/A	N/A	8.78E+01	N/A
DS-Cav1	RT	N/A	1.57E+05	N/A	N/A	7.15E+04	N/A	2.85E-05<br>N/A	5.10E+04<br>3.26E+05	5.60E-10<br>N/A	N/A	4.93E+04	N/A
		N/A	1.82E+05	N/A	N/A	9.65E+04	N/A	6.09E-04<br>N/A	5.93E+04<br>3.96E+05	1.03E-08<br>N/A	N/A	5.10E+04	N/A
	65	N/A	3.48E+04	N/A	N/A	1.05E+03	N/A	N/A	N/A	N/A	N/A	6.47E+01	N/A
		N/A	3.70E+04	N/A	N/A	5.34E+03	N/A	N/A	N/A	N/A	N/A	7.38E+01	N/A
	70	N.B.	N.B.	N.B.	N/A	1.39E+02	N/A	N/A	N/A	N/A	N.B.	N.B.	N.B.
		N.B.	N.B.	N.B.	N/A	5.85E+04	N/A	N/A	N/A	N/A	N.B.	N.B.	N.B.
R-1b	RT	N/A	1.82E+05	N/A	9.67E-06	1.36E+05	7.13E-11	2.91E-03<br>N/A	5.85E+04<br>5.00E+05	4.98E-08<br>N/A	N/A	6.60E+04	N/A
		N/A	1.82E+05	N/A	1.16E-05	1.22E+05	9.51E-11	3.40E-03<br>N/A	6.26E+04<br>5.72E+05	5.42E-08<br>N/A	N/A	5.58E+04	N/A

RT= Room Temperature, N.B. = No Binding. N/A= Not Applicable.
* Binding was calculated with a 2:1 binding model. The model assumes two molecules bind with different binding constants. Both constants are presented.

Supplementary Table 2. Binding kinetics of RSV F variants following exposure to various storage conditions, measured by bio-layer interferometry.

Protein Variants	Storage conditions	Antibodies								
		D25			hRSV90			MPE8		
		koff (1/s)	kon (1/Ms)	KD (M)	koff (1/s)	kon (1/Ms)	KD (M)	koff (1/s)	kon (1/Ms)	KD (M)
SsA1	25°C for 1 week	0.000016	1.61E+05	9.9E-11	0.000268	128000	2.09E-09	N/A	5.09E+04	N/A
		6.58E-06	1.59E+05	4.14E-11	0.000232	137000	1.69E-09	N/A	5.23E+04	N/A
	5°C for 1 month	N/A	1.88E+05	N/A	0.000186	137000	1.36E-09	N/A	5.51E+04	N/A
		N/A	1.84E+05	N/A	0.000162	149000	1.09E-09	N/A	6.47E+04	N/A
	-20°C for 1 month	N/A	1.97E+05	N/A	0.000195	142000	1.37E-09	N/A	53400	N/A
		N/A	1.92E+05	N/A	0.000137	138000	9.97E-10	N/A	63100	N/A
	-70°C for >1 year	0.0000429	1.76E+05	2.43E-10	0.000151	159000	9.53E-10	N/A	58200	N/A
		N/A	1.85E+05	N/A	0.000125	172000	7.29E-10	N/A	66200	N/A
DS-Cav1	25°C for 1 week	N/A	3.29E+05	N/A	N/A	131000	N/A	N/A	8.22E+04	N/A
		N/A	3.20E+05	N/A	N/A	148000	N/A	N/A	8.68E+04	N/A
	5°C for 1 month	N/A	1.41E+05	N/A	N/A	86800	N/A	N/A	4.61E+04	N/A
		N/A	1.43E+05	N/A	N/A	98100	N/A	N/A	4.62E+04	N/A
	-20°C for 1 month	N/A	1.57E+05	N/A	N/A	100000	N/A	N/A	49600	N/A
		N/A	1.66E+05	N/A	N/A	108000	N/A	N/A	55400	N/A
	-70°C for >1 year	N/A	1.70E+05	N/A	N/A	112000	N/A	N/A	54000	N/A
		N/A	1.72E+05	N/A	N/A	108000	N/A	N/A	53400	N/A
R-1b	25°C for 1 week	0.0000134	1.63E+05	8.18E-11	0.0000382	124000	3.08E-10	N/A	5.61E+04	N/A
		0.0000047	1.61E+05	2.92E-11	0.0000694	134000	5.16E-10	N/A	6.03E+04	N/A
	5°C for 1 month	N/A	1.86E+05	N/A	0.0000737	127000	5.81E-10	N/A	6.02E+04	N/A
		N/A	1.90E+05	N/A	0.0000514	141000	3.64E-10	N/A	6.88E+04	N/A

	-20°C for 1 month	N/A	1.96E+05	N/A	0.0000653	138000	4.74E-10	N/A	62400	N/A
		N/A	1.99E+05	N/A	0.0000243	133000	1.82E-10	N/A	61200	N/A
	-70°C for >1 year	N/A	1.95E+05	N/A	0.0000729	154000	4.73E-10	N/A	66200	N/A
		N/A	1.99E+05	N/A	0.000045	150000	3E-10	N/A	66400	N/A

References

[1] J. S. McLellan *et al.*, “Structure-Based Design of a Fusion Glycoprotein Vaccine for Respiratory Syncytial Virus,” *Science*, vol. 342, no. 6158, p. 592, Nov. 2013, doi: 10.1126/SCIENCE.1243283.

[2] Y. Che *et al.*, “Rational design of a highly immunogenic prefusion-stabilized F glycoprotein antigen for a respiratory syncytial virus vaccine,” *Sci. Transl. Med.*, vol. 15, no. 693, p. eade6422, 2023, doi: 10.1126/scitranslmed.ade6422.
